# Immune Cell Infiltration Characteristics of Pigmented Villous Nodular Synovitis and Prediction of Potential Diagnostic Markers Based on Bioinformatics

**DOI:** 10.1155/2022/8708692

**Published:** 2022-06-07

**Authors:** Jun Zhang, Bin Li, Boming Zhao, Yongjian Qi, Liaobin Chen, Jun Chen, Biao Chen

**Affiliations:** ^1^Division of Joint Surgery and Sports Medicine, Department of Orthopedic Surgery, Zhongnan Hospital of Wuhan University, Wuhan, 430000 Hubei, China; ^2^Department of Spine Surgery and Musculoskeletal Tumor, Department of Orthopedic Surgery, Zhongnan Hospital of Wuhan University, Wuhan, 430000 Hubei, China

## Abstract

**Background:**

Pigmented villous nodular synovitis (PVNS) is a tumor-like proliferative disease characterized by impairment of daily activities, decreased quality of life, and a high recurrence rate. However, the specific pathological mechanisms are still ill-defined and controversial. The purpose of this study was to define potential diagnostic markers and evaluate immune cell infiltration in the pathogenesis of PVNS.

**Method:**

The expression profile of GSE3698 was reanalyzed in the Gene Expression Omnibus (GEO) database. First, differentially expressed genes (DEGs) were identified using the R package “limma” and analyzed by Gene Ontology (GO) functional annotation and Kyoto Encyclopedia of Genes and Genomes (KEGG) pathway enrichment. Next, the DEGs were imported into the STRING database and Cytoscape to construct a protein–protein interaction (PPI) network. Then, cytoHubba and ROC curve analyses were used to determine potential diagnostic biomarkers of PVNS. Finally, we used CIBERSORT to estimate the proportions of 22 immune cell subtypes in PVNS and analyzed the correlation between diagnostic markers and infiltrating immune cells.

**Result:**

We found 139 DEGs (including 93 upregulated genes and 46 downregulated genes). TYROBP, FCER1G, LAPTM5, and HLA-DPB1 were identified as potential diagnostic biomarkers of PVNS. Immune cell infiltration analysis indicated that neutrophils and M2 macrophages might be associated with the genesis and progression of PVNS. Furthermore, our correlation analysis of diagnostic markers and infiltrating immune cells found that TYROBP, FCER1G, LAPTM5, and HLA-DPB1 were positively correlated with M2 macrophage infiltration and that neutrophils, TYROBP, FCER1G, and LAPTM5 were negatively correlated with plasma cell infiltration.

**Conclusions:**

We identified TYROBP, FCER1G, LAPTM5, and HLA-DPB1 as potential diagnostic markers for PVNS and concluded that immune cell infiltration plays an important role in the genesis and progression of PVNS.

## 1. Introduction

Pigmented villous nodular synovitis (PVNS) is a relatively rare, tumor-like proliferative disease in the synovium of the joint tissue [1]. PVNS favors the knee and hip, mainly affecting young adults between 20 and 40 years, with an incidence estimated to be between 1.8 per million. Clinically, PVNS manifests as a locally destructive process, leading to functional decline and decreased quality of life [2–4]. Moreover, the local recurrence rate is still high even after complete surgical resection, especially for the diffuse type [5]. If not treated in time, PVNS will lead to joint degenerative changes, moderate to severe joint deformity, and cortical bone destruction. Eventually, patients will require total joint arthroplasty or even amputation [6]. Currently, the primary therapeutic approach is surgical resection of the entire pathological synovial tissue to decrease joint pain and destruction, followed by postoperative chemotherapy to reduce the risk of recurrence [7]. However, it is still controversial whether PVNS is an inflammatory reaction or a neoplastic hyperplasia disease [8, 9]. Considering the high recurrence rate, extremely aggressive invasion, and destruction caused by PVNS, it is crucial to elucidate the molecular mechanisms, understand the underlying pathological mechanisms, and identify novel early diagnostic markers.

Previous studies have shown that the proportions of T cells, natural killer (NK) cells, and natural killer T (NKT) cells are significantly increased in the peripheral blood of PVNS patients. In addition, a significant increase in osteoclastogenesis and macrophage activation was observed in the local synovial membrane [10]. Additional studies have also suggested that macrophages and macrophage-like cells are involved in the occurrence and development of PVNS [11]. West et al. [12] showed that a minority chromosome involving the colony-stimulating factor 1 (CSF1) gene ectopic in synovial tissue resulted in the accumulation of monocytes and macrophages. These observations indicated that infiltrating immune cells might play an essential role in the pathological processes of PVNS. Therefore, it is critical to evaluate whether there is immune cell infiltration in PVNS and determine the intrinsic differences in infiltrating immune cell types for PVNS from an immune system perspective, which could help elucidate the potential mechanism of disease and suggest possible novel immunotherapy targets. With the rapid development of science and technology, innovative bioinformatic approaches have been developed that offer a powerful platform to look for potential diagnostic markers and evaluate immune cell infiltration [13]. CIBERSORT is a popular analytical tool that utilizes gene expression-profiling datasets to assess the expression of immune cells and analyze various immune cell proportions in the synovial tissue of PVNS and osteoarthritis (OA) [14]. At present, CIBERSORT has been widely used for research in myocarditis [15], breast cancer [13], atherosclerosis-related cardiovascular diseases [16], and many other conditions. However, to our knowledge, no studies on the use of CIBERSORT to analyze immune cell infiltration in PVNS have been reported thus far.

In this study, we first downloaded the original microarray dataset from the Gene Expression Omnibus (GEO) database and identified the differentially expressed genes implicated in PVNS. In addition, Gene Ontology (GO) functional enrichment and Kyoto Encyclopedia of Genes and Genomes (KEGG) were used to investigate the biological function of DEGs. Subsequently, the STRING database, Cytoscape, and receiver operating characteristic (ROC) curves were further used to filter and identify potential diagnostic biomarkers in PVNS. Moreover, in this study, for the first time, we applied the CIBERSORT method to analyze the differences in 22 immune cell subsets in PVNS using gene expression profiles. Finally, we investigated the relationship between the identified diagnostic marker genes and infiltrating immune cells to help better understand the pathological immune mechanisms of PVNS.

## 2. Materials and Methods

### 2.1. Data Source and Data Preprocessing

We downloaded the GSE3698 RAW dataset from the Gene Expression Omnibus (GEO, http://www.ncbi.nlm.nih.gov/geo), which contained 11 cases of PVNS synovial tissue and 19 cases of OA synovial tissue. The gene annotation platform of the GSE3698 dataset was GPL3050. First, the data were preprocessed using the “limma” package of R/Bioconductor [17]. Subsequently, probes were annotated using an annotation platform file. Probe sets without known annotation, mapped to multiple Gene IDs, or did not map to any Gene ID were removed. In cases where a gene corresponds to a plurality of probe sets, the maximum value was used as the gene expression value. Eventually, 30 samples and 9647 genes were processed for subsequent analysis.

### 2.2. Differentially Expressed Gene (DEG) Identifications

The R package “limma” was used to screen the differentially expressed genes, with absolute log2 fold change (logFC) > 0.5 and *P* < 0.05 as threshold. Volcano plots were produced using the R package “ggplot2,” and heat maps were produced using the R package “pheatmap” for differentially expressed genes. According to the logFC values, the top 10 genes upregulated and downregulated were selected to construct the heat map.

### 2.3. Pathway Enrichment Analyses

Gene Ontology (GO) and Kyoto Encyclopedia of Genes and Genomes (KEGG) were performed using Database for Annotation, Visualization, and Integrated Discovery (DAVID) tool (version 6.8) [18]. The GO terms and KEGG pathways with *P* < 0.05 and count > 1 were considered statistically significant and visualized through the R package “Goplot.”

### 2.4. Protein-Protein Interaction (PPI) Network Construction and Hub Gene Identification

The Search Tool for Retrieving Interacting Genes (STRING), which contains a database of known and predicted protein-protein interactions (PPI), was used to construct PPI networks of differentially expressed genes (confidence score cutoff = 0.5) [19]. The gene clusters were then imported into Cytoscape (version 3.9.0.) to visualize the networks [20]. Three analysis methods were used to screen out the top 13 hub genes, respectively, namely, Degree, Maximum Neighborhood Component (MNC), and Maximal Clique Centrality (MCC) in the Cytoscape plugin cytoHubba [21]. In addition, we looked for shared Hub genes of selection between three analysis methods and were visualized by Venn diagrams in R package “VennDiagram.”

### 2.5. ROC Curve Verification of Hub Genes

A receiver operating characteristic (ROC) curve was drawn, and an area under the curve (AUC) was calculated in R package “pROC” according to gene expression profile data of hub genes. In general, we considered AUC values of >0.9 indicated high diagnostic performance [22].

### 2.6. Immune Cell Infiltration Analysis

First, the GSE3698 expression matrix data and LM22 matrix transform gene expression were uploaded into R (version 4.1.2); the source code of CIBERSORT and the LM22 matrix transform gene expression were obtained from the CIBERSORTx website (https://cibersortx.stanford.edu/). Filtered out samples with *P* > 0.05 and removed noninfiltrating immune cells. The differences in immune cell infiltration between PVNS and OA samples were obtained in R package “vioplot.” The proportion of infiltrating immune cells in the sample is plotted in R package “ggplot2,” and the R package “corrplot” draws a heat map of infiltrating immune cell correlations.

### 2.7. Correlation Analysis between Diagnostic Markers and Infiltrating Immune Cells

We performed Spearman correlation analysis between the expression matrix data of diagnostic markers and infiltrating immune cells. Data were visualized using the ggplot2 package.

## 3. Results

### 3.1. DEG Identifications

**Figure 1:**
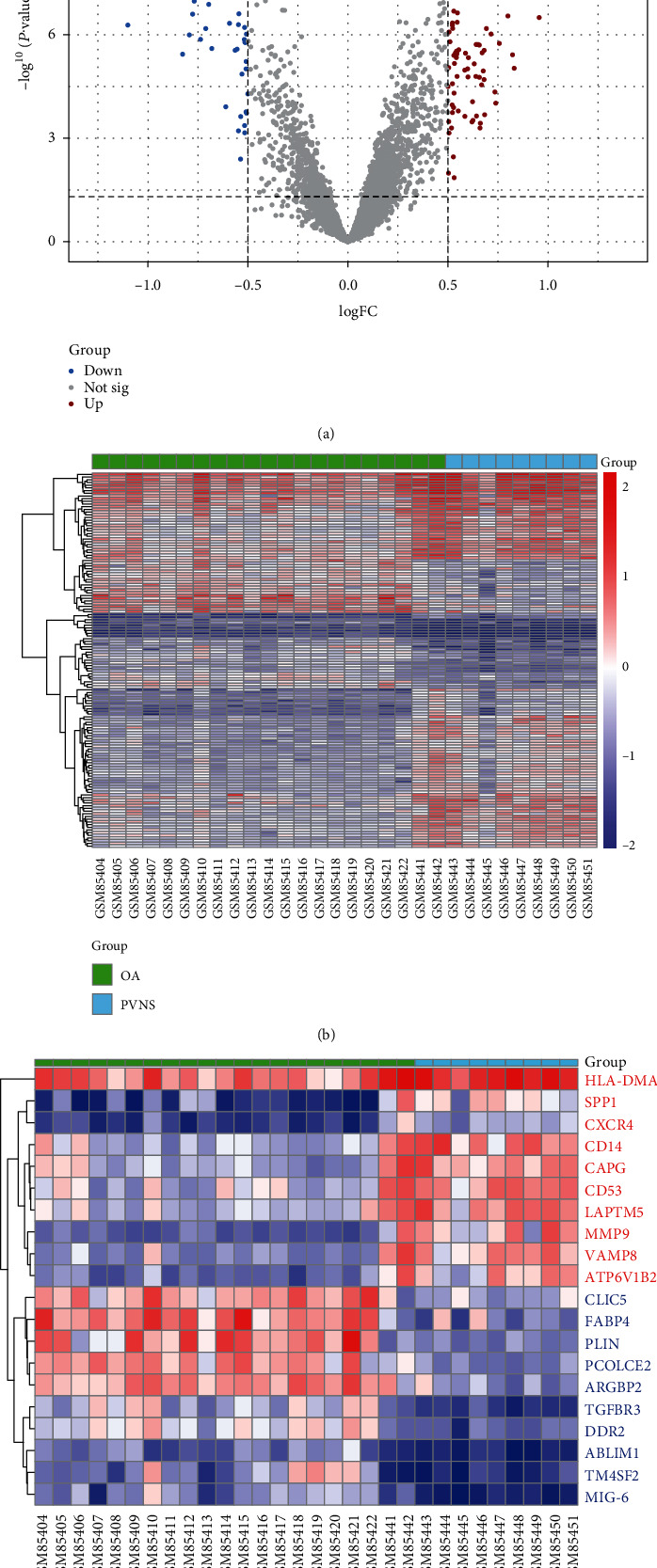
Volcano map and heat map of differentially expressed genes (DEGs). (a) Volcano map of DEGs, *P* < 0.05, and |logFC| > 0.5 as threshold; the upregulated and downregulated genes are represented by red and blue, respectively. (b) Heat map of DEGs. (c) Heat map of top 10 genes upregulated (red font) and downregulated (blue font) according to the logFC.

We identified a total of 139 DEGs (including 93 upregulated DEGs and 46 downregulated DEGs) in PVNS synovial tissue compared with OA synovial tissue, and a volcano plot is shown in Figure 1(a). A heat map of DEGs is shown in Figure 1(b). In addition, the heat map for the top 10 genes upregulated and downregulated according to the logFC values is shown in Figure 1(c).

### 3.2. Pathway Enrichment Analyses

**Figure 2:**
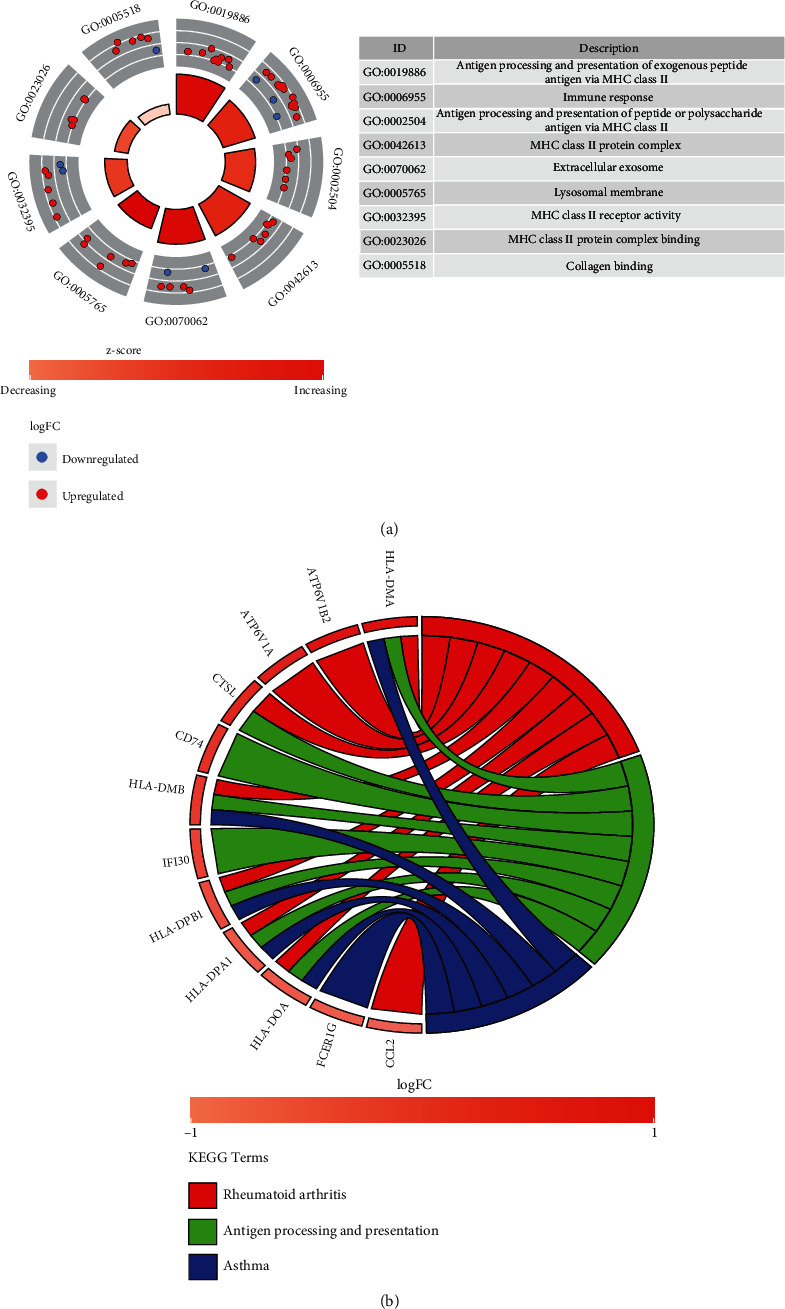
GO and KEGG enrichment analysis results of DEGs. (a) The circle diagram shows the enrichment results of DEGs and the top 9 GO pathways. Red dots represent upregulated genes, and blue dots represent downregulated genes. (b) Chord plot shows the association of DEGs with the first three KEGG enriched pathways; colors are represented by logFC.

We further performed GO and KEGG pathway enrichment analyses on DEGs using the DAVID database to explore the potential pathological process of PVNS. Functional enrichment analysis revealed that 44 biological process (BP), 30 cellular component (CC), 18 molecular function (MF) (Table S1), and 22 KEGG pathways (Table S2) were statistically significant. We screened out the top three GO-BP, GO-CC, and GO-MF pathways and drew a circle plot showing the number of genes enriched in each GO pathway (Figure 2(a)). The top three GO-BPs were antigen processing and presentation of exogenous peptide antigen via MHC class II, immune response, antigen processing, and presentation of peptide or polysaccharide antigen via MHC class II. The top three GO-CC terms were extracellular exosome, MHC class II protein complex, and lysosomal membrane; the top three GO-MF terms were MHC class II receptor activity, collagen binding, and MHC class II protein complex binding.  Figure 2(b) shows the top three KEGG pathways and the related genes enriched in each pathway. The top three KEGG pathways were rheumatoid arthritis, asthma, antigen processing, and presentation.

### 3.3. PPI Network Construction and Hub Gene Identification

**Figure 3:**
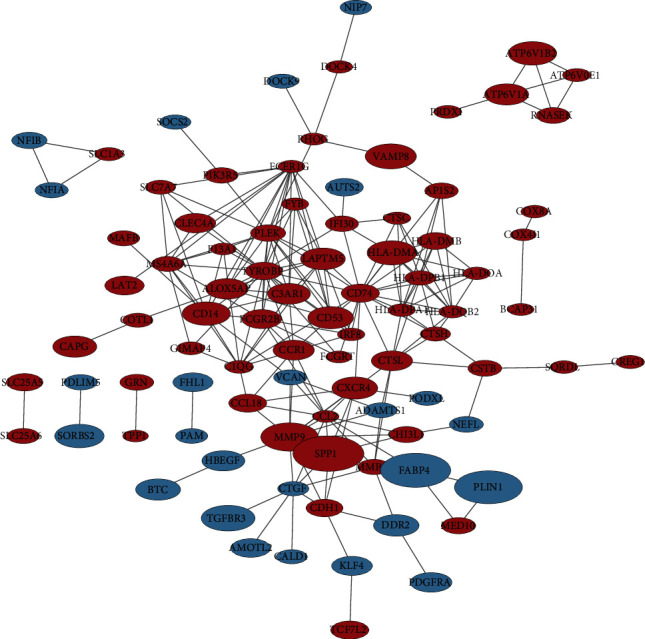
PPI network of DEGs; blue represents downregulated genes, red represents upregulated genes, and size represents logFC.

**Figure 4:**
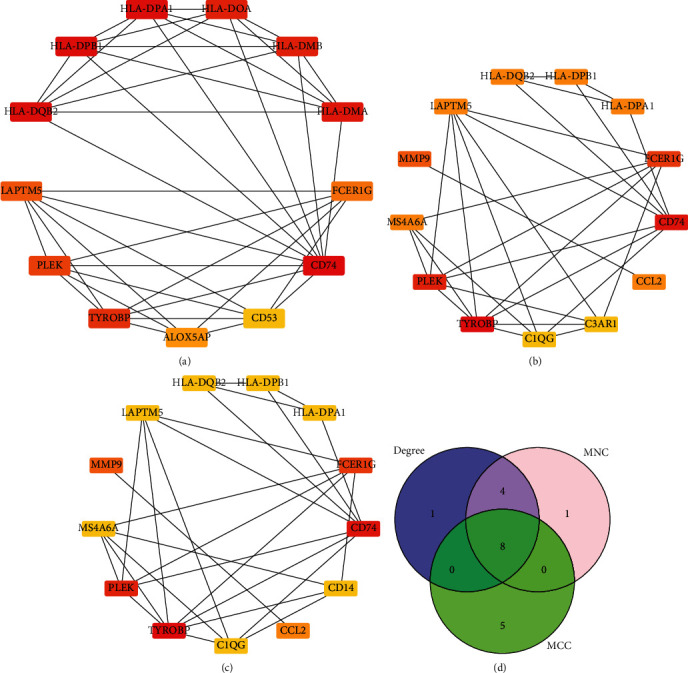
(a) Gene set network diagram of MCC algorithm in cytoHubba, the redder the color, the higher the ranking, and the yellower the color, the lower the ranking. (b) The gene set network diagram of the MNC algorithm in cytoHubba, the redder the color, the higher the ranking, and the yellower the color, the lower the ranking. (c) The gene set network diagram of the degree algorithm in cytoHubba, the redder the color in the box, the higher the ranking, and the yellower the color, the lower the ranking. (d) Venn diagrams of gene sets obtained from the three algorithms.

We uploaded 139 DEGs into the STRING database to construct PPI networks visualized by the Cytoscape software. The PPI network showed 90 nodes, where each node represents a DEG, including 25 upregulated genes and 65 downregulated genes (Figure 3). The top 13 genome modules were obtained from the PPI network through three methods: MCC, MNC, and degree in the cytoHubba plugin of the Cytoscape software. We screened 8 hub genes (TYROBP, FCER1G, LAPTM5, HLA-DPB1, CD74, HLA-DPA1, HLA-DQB2, PLEK) with upregulated expression by taking the intersection of genome modules (Figure 4).

### 3.4. ROC Curve Verification of Hub Genes

**Figure 5:**
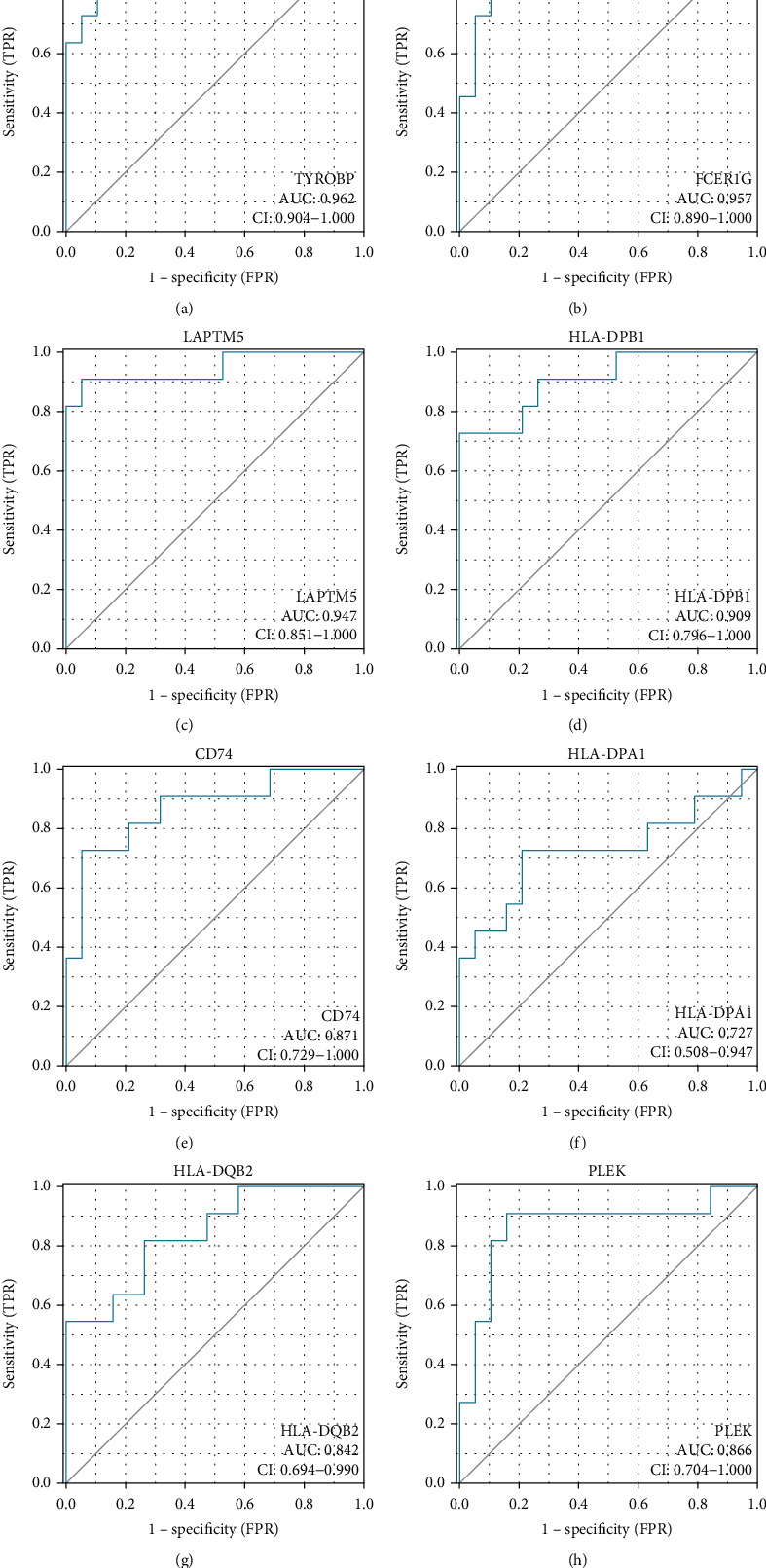
ROC prediction curve in hub gene: (a) TYROBP; (b) FCER1G; (c) LAPTM5; (d) HLA-DPB1; (e) CD74; (f) HLA-DPA1; (g) HLA-DQB2; (h) PLEK. ROC: receiver operating curve analysis.

We then assessed the diagnostic sensitivity of the hub genes by ROC curve analysis. The area under the curve (AUC) for TYROBP was 0.962 (95% confidence interval (CI): 0.904-1.000) (Figure 5(a)); the area under the curve (AUC) for FCER1G was 0.957 (95% confidence interval (CI): 0.890-1.000) (Figure 5(b)); the area under the curve (AUC) for LAPTM5 was 0.947 (95% confidence interval (CI): 0.851-1.000) (Figure 5(c)); the area under the curve (AUC) for HLA-DPB1 was 0.909 (95% confidence interval (CI): 0.796-1.000) (Figure 5(d)); the area under the curve (AUC) for CD74 was 0.871 (95% confidence interval (CI): 0.729-1.000) (Figure 5(e)); the area under the curve (AUC) for HLA-DPA1 was 0.727 (95% confidence interval (CI): 0.508-0.957) (Figure 5(f)); the area under the curve (AUC) for HLA-DQB2 was 0.842 (95% confidence interval (CI): 0.694-0.990) (Figure 5(g)); the area under the curve (AUC) for PLEK was 0.866 (95% confidence interval (CI): 0.704-1.000) (Figure 5(h)). Based on the high diagnostic value of AUC > 0.9 [22], we identified TYROBP, FCER1G, LAPTM5, and HLA-DPB1 as potential diagnostic markers in PVNS.

### 3.5. Immune Cell Infiltration Analysis

**Figure 6:**
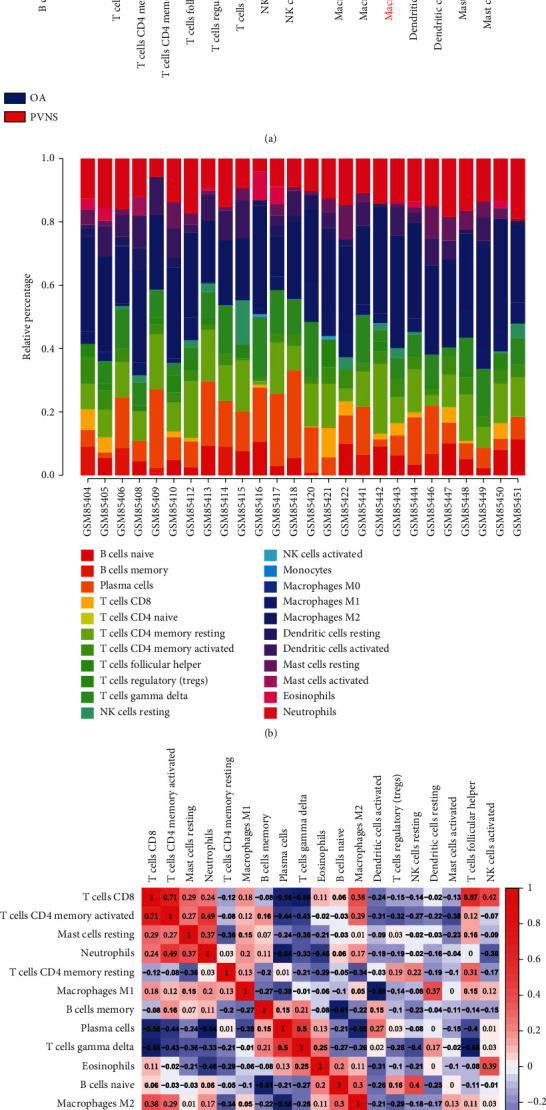
Assessment and visualization of immune cell infiltration. (a) Violin plot of the proportion of 22 immune cells, with red markers representing significant infiltration differences between the two groups. (b) The proportion of 22 immune cells between-sample. (c) Correlation heat map of 19 types of immune cells. The size of the colored square numbers represents the strength of the correlation; red represents positive correlation, and blue represents negative correlation. The darker the color, the stronger the correlation.

A violin plot of differences in immune cell infiltration suggested that 19 types of immune cell infiltration were present in PVNS samples. At the same time, the infiltration of monocytes, naive CD4+ T cells, and M0 macrophages was not detected in PVNS. M2 macrophages (*P* = 0.017) and neutrophils (*P* = 0.047) were expressed at significantly higher levels in PVNS than in OA (Figure 6(a)).  Figure 6(b) shows the individual sample's proportion of infiltrated immune cells. A correlation heat map of the 19 types of infiltrating immune cells revealed that regulatory T cells had the greatest positive correlation with resting mast cells (*r* = 0.88). CD8+ T cells had a positive correlation with activated CD4+ memory T cells (*r* = 0.71). Resting dendritic cells had a positive correlation with activated mast cells (*r* = 0.62). M1 macrophages had the greatest negative correlation with activated dendritic cells (*r* = −0.68). Gamma-delta T cells had a negative correlation with CD8+ T cells (*r* = −0.66) and follicular helper T cells (*r* = −0.66). Plasma cells had a negative correlation with neutrophils (*r* = −0.64), M2 macrophages (*r* = −0.58), and CD8+ T cells (*r* = −0.56). Naive B cells had a negative correlation (*r* = −0.61) with memory B cells (Figure 6(c)).

### 3.6. Correlation Analysis between Diagnostic Markers and Infiltrating Immune Cells

**Figure 7:**
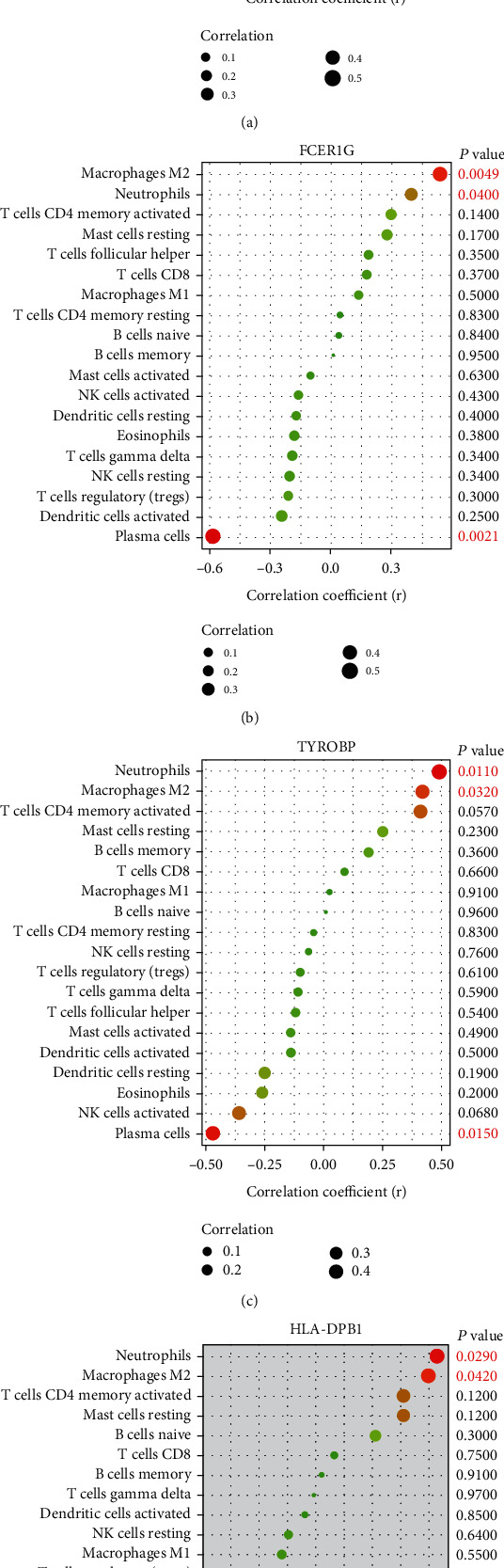
Correlation between LAPTM5, FCER1G, TYROBP, and HLA-DPB1 with infiltrating immune cells: (a) correlation between LAPTM5 and infiltrating immune cells; (b) correlation between FCER1G and infiltrating immune cells; (c) correlation between TYROBP and infiltrating immune cells; (d) correlation between HLA-DPB1 and infiltrating immune cells. The size of the dot represents the strength of the correlation between genes and immune cells; the larger the dot, the stronger the correlation, and the smaller the dot, the weaker the correlation. The color of the point represents the *P* value; the greener the color, the larger the *P* value, and the redder the color, the smaller the *P* value. *P* < 0.05 was considered statistically significant.

A correlation analysis was performed among four diagnostic markers, TYROBP, FCER1G, LAPTM5, and HLA-DPB1, and 19 types of infiltrating immune cells. The results showed that LAPTM5 was positively correlated with M2 macrophages (*r* = 0.53, *P* = 0.0058) and neutrophils (*r* = 0.43, *P* = 0.0027) and negatively correlated with plasma cells (*r* = −0.5, *P* = 0.0097); FCER1G was positively correlated with M2 macrophages (*r* = 0.54, *P* = 0.0049) and neutrophils (*r* = 0.4, *P* = 0.04) and negatively correlated with plasma cells (*r* = −0.58, *P* = 0.0021); TYROBP was positively correlated with M2 macrophages (*r* = 0.49, *P* = 0.011) and neutrophils and negatively correlated with plasma cells (*r* = −0.47, *P* = 0.015); HLA-DPB1 was positively correlated with M2 macrophages (*r* = 0.43, *P* = 0.029) and neutrophils (*r* = 0.4, *P* = 0.042) (Figure 7).

## 4. Discussion

PVNS is a relatively rare tumor-like proliferative disease of the knee joint. As the disease progresses, local manifestations include artificial knee effusions, hemosiderin deposition, synovial hyperplasia, and bone erosions. Moderate to severe joint destruction and stiffness due to recurrent hemarthrosis may also occur [2]. If the articular cartilage is severely eroded, cortical bone destruction will necessitate total synovectomy, total knee replacement, or even amputation. Moreover, patients typically relapse after discontinuation of therapy [23]. PVNS progression is usually insidious and begins many years before the onset of clinical symptoms. It will cause a substantial economic and psychological burden to the patient.

The GSE3698 dataset was downloaded from the GEO database, and 139 DEGs were identified. GO analysis revealed that these genes were mainly involved in antigen processing and presentation of exogenous peptide antigens via MHC class II and the immune response. KEGG analysis revealed that these genes were mainly involved in rheumatoid arthritis, asthma, and antigen processing and presentation. The above results suggested that inflammation and immunological reactions might play an essential role in PVNS. Zhao et al. confirmed that increased immune cell infiltration and cytokine secretion in PVNS synovial tissue affected its pathological process [10]. Cao et al. [24] found that inflammatory factors were significantly upregulated in PVNS knee synovial fluid. Therefore, our conclusions are consistent with the above reports.

Cytoscape is an open-source software project for integrating biomolecular interaction networks with high-throughput expression data and other molecular states into a unified conceptual framework [20]. The cytoHubba plugin ranks nodes with various algorithms based on network characteristics [21]. In this study, based on three of these algorithms, we identified TYROBP, CD74, PLEK, HLA-DPA1, HLA-DQB2, LAPTM5, HLA-DPB1, and FCER1G as the hub genes of PVNS. ROC analysis is a tool for evaluating model accuracy and has been commonly used for disease screening, diagnosis, treatment, and prognosis [22]. We further predicted the diagnostic value of 8 hub genes using ROC curves. The results showed that TYROBP, FCER1G, LAPTM5, and HLA-DPB1 could be used as high-value diagnostic markers for PVNS.

TYROBP is a type I transmembrane protein, also known as DAP12, consisting of a leader peptide, cysteine residues, a transmembrane fragment of aspartic acid residues, and a cytoplasmic tyrosine activation motif containing an immunoreceptor domain [25]. TYROBP is expressed in immune cells such as macrophages, monocytes, and osteoclasts [26]. Studies have shown that upregulated TYROBP might promote osteoporosis through osteoclasts [27]. Given that osteoclasts are essential in the pathological development of PVNS [28], we believe that TYROBP upregulation might be involved in the pathological progression of PVNS by regulating osteoclast differentiation. FCER1G is located on chromosome 1q23 and the gamma subunit of immunoglobulin E (IgE) encoding the crystallizable fragment (Fc) region [29]. Early studies found that various immune receptor activation signals transduced by FCER1G were particularly important in chronic responses such as autoimmunity and chronic infection [30]. FCER1G recognizes and eliminates non-self-antigens in normal immune system states but may trigger destructive inflammation, immune cell activation, phagocytosis, oxidative burst, and cytokine release in pathological conditions [31]. Furthermore, Dang et al. [32] discovered that FCER1G might be an ideal candidate for a robust, universal, and optimal marker of both macrophages and immune system players. Considering the macrophage accumulation in the synovial tissue PVNS, we believe that high expression of FCER1G might be involved in PVNS inflammation and immune pathways through macrophages. LAPTM5, also known as E3 protein, is a lysosomal membrane protein preferentially expressed in immune cells that interacts with the Nedd4 family of ubiquitin ligases, which play a role in hematopoiesis by preventing lymphocyte hyperactivation [33]. LAPTM5 is a positive regulator of the inflammatory signaling cascade in macrophages, and low expression of LAPTM5 inhibits the bladder cancer cell cycle [34, 35]. The above studies indicated that high expression of LAPTM5 might participate in the cascade regulation of the inflammatory signaling pathway, regulate the cell cycle, and stimulate cell hyperproliferation in PVNS. HLA-DPB1, located in the HLA class II region, is present on the cell surface of antigen-presenting cells. Genetic variants in HLA-DPB1 are associated with various autoimmune diseases, including rheumatoid arthritis, Graves' disease, and multiple sclerosis [36]. However, whether HLA-DPB1 is associated with PVNS remains unclear.

To further explore the pathological process of immune cell infiltration in PVNS, we performed a comprehensive assessment using CIBERSORT. It is noteworthy that we used OA synovium as the control group. A certain degree of immune infiltration changes was also observed in the OA synovium. Some studies reported that, compared with normal synovium, there was no significant difference in M2 macrophages and neutrophils in OA synovium [37, 38]. Our results suggested that the infiltration of M2 macrophages and neutrophils was significantly increased in PVNS synovial tissue compared to OA synovial tissue. Our results further suggested that these two types of immune cell infiltration might play an essential role in the pathogenesis of PVNS. Previous studies have shown abnormal proliferation of macrophages and high expression of macrophage markers in the pathological process of PVNS [11]. Another study has shown that synovial tissue in PVNS abnormally secretes colony-stimulating factor 1, which recruits macrophages through binding to colony-stimulating factor 1 receptor to abnormally accumulate and locally form tumor-like masses, which constitute most of the tumor cell population and present as a tumor landscape effect in PVNS synovium [12]. In addition, the recruited macrophages could be polarized to the M2 phenotype [39, 40]. It has been reported that tumor-associated macrophages display an M2 macrophage phenotype, which can promote tumor growth and angiogenesis and invade local tissues. Therefore, we speculate that the highly infiltrated M2 macrophages in the PVNS synovium might be involved in mass growth and local bone erosion. Neutrophils can induce RANKL (receptor activator of NF-*κ*B ligand) expression and induce osteoclast formation [41, 42]. Several studies have shown that a large number of osteoclast-type giant cells in PVNS synovial tissue could affect local bone remodeling, further leading to osteolysis and cortical bone destruction [10, 28]. Therefore, we believe that the highly infiltrated neutrophils in the PVNS synovium might lead to local osteolysis and cortical bone destruction through osteoclasts. Additionally, we revealed details of the correlation of immune cell infiltration and found that regulatory T cells had the greatest positive correlation with resting mast cells. CD8+ T cells had a positive correlation with activated CD4+ memory T cells. Resting dendritic cells had a positive correlation with activated mast cells. M1 macrophages had the greatest negative correlation with activated dendritic cells. Gamma-delta T cells had a negative correlation with CD8+ T cells and follicular helper T cells. Plasma cells had a negative correlation with neutrophils, M2 macrophages, and CD8+ T cells. Naive B cells had a negative correlation with memory B cells.

The correlation analysis displayed that FCER1G, LAPTM5, TYROBP, and HLA-DPB1 were positively correlated with M2 macrophages and neutrophils. FCER1G, LAPTM5, and TYROBP were negatively correlated with plasma cells. Combined with the above results, these results suggested that the high expression of FCER1G, LAPTM5, TYROBP, and HLA-DPB1 might promote the recruitment, differentiation, and proliferation of macrophages and neutrophils and further participate in the occurrence and development of PVNS. The effects of plasma cells on PVNS have not been studied thus far. It has been reported that plasma cells can produce a large number of cytokines and antibodies during tumor infiltration [43]. These antibodies could further promote antitumor immunity by driving antibody-dependent cellular cytotoxicity (ADCC), phagocytosis, and complement activation and enhancing antigen presentation by dendritic cells [44]. In addition, in this study, we found that plasma cells were significantly negatively correlated with neutrophils and M2 macrophages through correlation analysis. Therefore, we inferred that high expression of FCER1G, LAPTM5, and TYROBP might weaken the antitumor protective effect of plasma cells by reducing plasma cell infiltration. Our inferences require further experimental studies to demonstrate the interaction between the hub genes and immune cells infiltrated in PVNS.

However, our research has certain limitations. First, the samples in GSE3698 are unpaired, so more paired sample data are needed to validate our conclusions. Second, although the CIBERSORT analysis showed lower estimation bias than other methods, it was based on limited genetic data, which might still bias the final results. Third, the function and immune cell infiltration of the four biomarkers in PVNS were inferred by bioinformatics analysis. An experimental study with a larger sample size should be performed to validate our conclusions.

## 5. Conclusions

In this study, we found for the first time that LAPTM5, FCER1G, TYROBP, and HLA-DPB1 might be novel diagnostic markers for PVNS. In addition, for the first time, the infiltration of immune cells in the pathological process of PVNS was identified by CIBERSORT, and we found that M2 macrophages and neutrophils might be involved in the immune regulation process of PVNS. LAPTM5, FCER1G, TYROBP, and HLA-DPB1 were positively correlated with M2 macrophage and neutrophil infiltration, and LAPTM5, FCER1G, and TYROBP were negatively associated with plasma cell infiltration. These abnormally expressed genes and immune cells might play vital roles in the pathological process of PVNS. Further exploration of the regulatory interaction between these genes and immune cells will provide new clinical diagnostic markers and therapeutic targets for PVNS.

## Data Availability

The data used to support the findings of this study are available from the GEO database (https://www.ncbi.nlm.nih.gov/geo/).

## Supplementary Materials

Supplementary Materials See Tables S1-S2 in Supplementary Materials for all pathway enrichment analyses.
