# Supplementary material for: Immune Cell Infiltration Characteristics of Pigmented Villous Nodular Synovitis and Prediction of Potential Diagnostic Markers Based on Bioinformatics
Source: Biomed Res Int. 2022 Jun 7;2022:8708692. doi: 10.1155/2022/8708692 (PMC9197622; doi:10.1155/2022/8708692)
Supplement: Supplementary Materials — See Tables S1-S2 in Supplementary Materials for all pathway enrichment analyses. [file 8708692.f1.docx]

## **Supplementary Materials**

Table S1: GO analysis of DEGs associated with PVNS

| **Ontology** | **ID** | **GO Term** | **P value** | **Genes** |  |
| --- | --- | --- | --- | --- | --- |
| BP | GO:0019886 | antigen processing and presentation of exogenous peptide antigen via MHC class II | 4.04E-10 | CD74, HLA-DMA, HLA-DMB, FCER1G, CTSL, AP1S2, HLA-DPB1, IFI30, HLA-DOA, HLA-DQB2, HLA-DPA1 |  |
| BP | GO:0006955 | immune response | 1.30E-08 | CCR1, CD74, SEMA3C, FYB, TGFBR3, HLA-DMA, HLA-DMB, C7, HLA-DPB1, IRF8, CCL2, FCGR2B, HLA-DOA, CCL18, HLA-DQB2, CTSC, HLA-DPA1 |  |
| BP | GO:0002504 | antigen processing and presentation of peptide or polysaccharide antigen via MHC class II | 6.11E-08 | HLA-DMA, HLA-DMB, HLA-DPB1, HLA-DOA, HLA-DQB2, HLA-DPA1 |  |
| BP | GO:0019882 | antigen processing and presentation | 1.46E-06 | CD74, HLA-DMB, FCGRT, CTSL, HLA-DPB1, CTSH, HLA-DPA1 |  |
| BP | GO:0060326 | cell chemotaxis | 6.37E-05 | PDGFRA, DOCK4, RHOG, C3AR1, CCL2, HBEGF |  |
| BP | GO:0022617 | extracellular matrix disassembly | 1.35E-04 | MMP13, CDH1, CTSL, SPP1, CAPG, MMP9 |  |
| BP | GO:0070374 | positive regulation of ERK1 and ERK2 cascade | 9.82E-04 | CCR1, PDGFRA, CD74, CCL2, CHI3L1, CCL18, CTGF |  |
| BP | GO:0060333 | interferon-gamma-mediated signaling pathway | 0.001169 | HLA-DPB1, IRF8, IFI30, HLA-DQB2, HLA-DPA1 |  |
| BP | GO:0050900 | leukocyte migration | 0.001193 | CD74, SLC7A7, FCER1G, PODXL, C3AR1, MMP9 |  |
| BP | GO:0030335 | positive regulation of cell migration | 0.001271 | CCR1, PDGFRA, SEMA3C, PODXL, SEMA3B, CTSH, HBEGF |  |
| BP | GO:0032729 | positive regulation of interferon-gamma production | 0.003297 | HLA-DPB1, IRF8, CD14, HLA-DPA1 |  |
| BP | GO:0050885 | neuromuscular process controlling balance | 0.003297 | CLIC5, SLC1A3, NEFL, TPP1 |  |
| BP | GO:0007187 | G-protein coupled receptor signaling pathway, coupled to cyclic nucleotide second messenger | 0.003297 | CCR1, NPY1R, CCL2, SSTR1 |  |
| BP | GO:0007229 | integrin-mediated signaling pathway | 0.003942 | TYROBP, FCER1G, ADAMTS1, PLEK, CTGF |  |
| BP | GO:0048146 | positive regulation of fibroblast proliferation | 0.005189 | BTC, PDGFRA, CD74, DDR2 |  |
| BP | GO:0050870 | positive regulation of T cell activation | 0.005969 | HLA-DPB1, CCL2, HLA-DPA1 |  |
| BP | GO:0009611 | response to wounding | 0.007966 | CCR1, SLC1A3, CCL2, CTGF |  |
| BP | GO:0006935 | chemotaxis | 0.008212 | CCR1, C3AR1, CCL2, CXCR4, CCL18 |  |
| BP | GO:0070098 | chemokine-mediated signaling pathway | 0.011046 | CCR1, CCL2, CXCR4, CCL18 |  |
| BP | GO:0007204 | positive regulation of cytosolic calcium ion concentration | 0.011319 | BCAP31, CCR1, PDGFRA, C3AR1, CXCR4 |  |
| BP | GO:0006954 | inflammatory response | 0.011781 | CCR1, SPP1, C3AR1, CCL2, CHI3L1, CXCR4, CD14, CCL18 |  |
| BP | GO:0042590 | antigen processing and presentation of exogenous peptide antigen via MHC class I | 0.012941 | FCER1G, IFI30 |  |
| BP | GO:0001503 | ossification | 0.015228 | SPP1, MMP9, CTGF, DDR2 |  |
| BP | GO:0051897 | positive regulation of protein kinase B signaling | 0.017335 | TCF7L2, CHI3L1, PIK3R5, HBEGF |  |
| BP | GO:0018108 | peptidyl-tyrosine phosphorylation | 0.017631 | BTC, PDGFRA, ABI2, DDR2, HBEGF |  |
| BP | GO:0046854 | phosphatidylinositol phosphorylation | 0.023284 | BTC, PDGFRA, PIK3R5, HBEGF |  |
| BP | GO:0038094 | Fc-gamma receptor signaling pathway | 0.025715 | FCER1G, FCGRT |  |
| BP | GO:0015853 | adenine transport | 0.025715 | SLC25A5, SLC25A6 |  |
| BP | GO:0002283 | neutrophil activation involved in immune response | 0.025715 | TYROBP, FCER1G |  |
| BP | GO:0006810 | transport | 0.025919 | ATP6V1A, CLIC5, SLC7A7, FABP4, LAPTM5, SLC25A5, SLC7A2 |  |
| BP | GO:0000165 | MAPK cascade | 0.028047 | BTC, PDGFRA, PAQR3, NEFL, CCL2, HBEGF |  |
| BP | GO:0043547 | positive regulation of GTPase activity | 0.030732 | BTC, PDGFRA, RGS5, DOCK4, DOCK9, NEFL, CCL2, CCL18, HBEGF |  |
| BP | GO:0098609 | cell-cell adhesion | 0.031784 | CALD1, DOCK9, PRDX1, CAPG, FLNB, PDLIM5 |  |
| BP | GO:0002503 | peptide antigen assembly with MHC class II protein complex | 0.032041 | HLA-DMA, HLA-DMB |  |
| BP | GO:0050920 | regulation of chemotaxis | 0.032041 | PDGFRA, CXCR4 |  |
| BP | GO:0007166 | cell surface receptor signaling pathway | 0.033096 | CD53, CCR1, NPY1R, CCL2, CD14, SSTR1 |  |
| BP | GO:0071356 | cellular response to tumor necrosis factor | 0.034822 | FABP4, CCL2, CHI3L1, CCL18 |  |
| BP | GO:0003151 | outflow tract morphogenesis | 0.035902 | TGFBR3, SEMA3C, NPY1R |  |
| BP | GO:0032760 | positive regulation of tumor necrosis factor production | 0.037342 | FCER1G, CCL2, CD14 |  |
| BP | GO:0007165 | signal transduction | 0.037662 | MPP1, CD53, CD74, GRN, UNC5C, FYB, TYROBP, CCL2, FLNB, FCGR2B, HLA-DOA, CCL18, HBEGF, DDR2 |  |
| BP | GO:1990822 | basic amino acid transmembrane transport | 0.038326 | SLC7A7, SLC7A2 |  |
| BP | GO:0051603 | proteolysis involved in cellular protein catabolic process | 0.038804 | CTSL, CTSH, CTSC |  |
| BP | GO:0043551 | regulation of phosphatidylinositol 3-kinase activity | 0.044571 | KLF4, PIK3R5 |  |
| BP | GO:0051549 | positive regulation of keratinocyte migration | 0.044571 | MMP9, HBEGF |  |
| CC | GO:0042613 | MHC class II protein complex | 3.61E-09 | CD74, HLA-DMA, HLA-DMB, HLA-DPB1, HLA-DOA, HLA-DQB2, HLA-DPA1 |  |
| CC | GO:0070062 | extracellular exosome | 5.16E-09 | ATP6V1A, CSTB, CLIC5, GRN, SEMA3C, PCOLCE2, COX4I1, SEMA3B, CXCR4, LTBP2, CAPG, HNMT, SLC2A5, HLA-DMA, SYNGR2, C7, CDH1, CTSL, PODXL, CREG1, PRDX1, SPP1, COTL1, CTSH, FLNB, CD14, CTSC, CD53, CD74, RHOG, MMP9, TGFBR3, VAMP8, MARCKS, FABP4, RNF149, ATP6V1B2, CHI3L1, TPP1, SLC25A5, PAM, SMPDL3A, ITM2A, DDR2 |  |
| CC | GO:0005765 | lysosomal membrane | 1.19E-06 | VAMP8, ATP6V1A, CD74, HLA-DMA, HLA-DMB, AP1S2, HLA-DPB1, LAPTM5, ATP6V1B2, HLA-DOA, HLA-DQB2, HLA-DPA1 |  |
| CC | GO:0030669 | clathrin-coated endocytic vesicle membrane | 5.49E-06 | CD74, AP1S2, HLA-DPB1, HLA-DQB2, HLA-DPA1, HBEGF |  |
| CC | GO:0005615 | extracellular space | 8.41E-06 | CSTB, GRN, SEMA3C, SEMA3B, LTBP2, MMP9, CTGF, TGFBR3, BTC, MMP13, CTSL, PODXL, CREG1, PRDX1, SPP1, CTSH, CCL2, CHI3L1, CD14, CCL18, PAM, SMPDL3A, CTSC, HBEGF |  |
| CC | GO:0071556 | integral component of lumenal side of endoplasmic reticulum membrane | 3.06E-05 | BCAP31, CD74, HLA-DPB1, HLA-DQB2, HLA-DPA1 |  |
| CC | GO:0005886 | plasma membrane | 8.55E-05 | ATP6V1A, DOCK4, FHL1, SLC40A1, SLC1A3, CXCR4, IFI30, SLC2A5, SLC7A2, FYB, CTGF, PIK3R5, RGS5, SV2B, CALD1, CDH1, FCGRT, PODXL, C3AR1, FLNB, CD14, HLA-DOA, HLA-DPA1, CD53, CCR1, CD74, PDGFRA, FCER1G, RHOG, NPY1R, UNC5C, SSTR1, VAMP8, BTC, MARCKS, TYROBP, SLC7A7, ATP6V1B2, HLA-DPB1, FCGR2B, PAM, ITM2A, HLA-DQB2, HBEGF, DDR2 |  |
| CC | GO:0005887 | integral component of plasma membrane | 1.67E-04 | MPP1, CD53, ATP6V1A, CCR1, BCAP31, PDGFRA, FCER1G, SLC40A1, NPY1R, LAPTM5, SSTR1, SLC2A5, SLC7A2, TGFBR3, SLC7A7, TYROBP, PODXL, C3AR1, SLC25A5, HLA-DPA1, HBEGF, DDR2 |  |
| CC | GO:0015629 | actin cytoskeleton | 4.44E-04 | CLIC5, MARCKS, ABLIM1, CALD1, CDH1, FLNB, PDLIM5, FYB |  |
| CC | GO:0009986 | cell surface | 5.91E-04 | CD53, TGFBR3, CD74, HLA-DMA, TYROBP, FCER1G, HLA-DPB1, SLC1A3, CXCR4, PAM, HLA-DPA1, HBEGF |  |
| CC | GO:0030666 | endocytic vesicle membrane | 7.76E-04 | CD74, HLA-DPB1, HLA-DQB2, HLA-DPA1, HBEGF |  |
| CC | GO:0005578 | proteinaceous extracellular matrix | 0.001486 | TGFBR3, MMP13, ADAMTS1, COL22A1, CHI3L1, LTBP2, MMP9, CTGF |  |
| CC | GO:0030658 | transport vesicle membrane | 0.001715 | CD74, HLA-DPB1, HLA-DQB2, HLA-DPA1 |  |
| CC | GO:0032588 | trans-Golgi network membrane | 0.001825 | CD74, AP1S2, HLA-DPB1, HLA-DQB2, HLA-DPA1 |  |
| CC | GO:0005764 | lysosome | 0.002985 | CTSL, LAPTM5, CTSH, CXCR4, TPP1, IFI30, CTSC |  |
| CC | GO:0043005 | neuron projection | 0.003769 | SV2B, SLC1A3, UNC5C, SSTR1, PDLIM5, HNMT, PAM |  |
| CC | GO:0012507 | ER to Golgi transport vesicle membrane | 0.004216 | CD74, HLA-DPB1, HLA-DQB2, HLA-DPA1 |  |
| CC | | GO:0016020 | membrane | 0.008871 | MPP1, BCAP31, PDGFRA, CD74, DOCK4, DOCK9, COX4I1, PLEK, SLC1A3, SLC7A2, PIK3R5, VAMP8, HLA-DMA, SV2B, RNF149, CDH1, FKBP1B, ALOX5AP, HLA-DPB1, SLC25A5, PDLIM5, PAM, CTSC, HTATIP2 |
| CC | GO:0032420 | stereocilium | 0.012294 | MPP1, CLIC5, DOCK4 |  |
| CC | GO:0043209 | myelin sheath | 0.015299 | ATP6V1A, PRDX1, ATP6V1B2, NEFL, SLC25A5 |  |
| CC | GO:0005913 | cell-cell adherens junction | 0.016002 | CALD1, DOCK9, CDH1, PRDX1, CAPG, FLNB, PDLIM5 |  |
| CC | GO:0043202 | lysosomal lumen | 0.016244 | CD74, CTSL, TPP1, IFI30 |  |
| CC | GO:0005829 | cytosol | 0.019705 | ATP6V1A, DOCK4, DOCK9, PLEK, FHL1, GIMAP4, NPL, HNMT, DTX4, FYB, CTGF, PIK3R5, SOCS2, FRAT1, CALD1, PRDX1, AP1S2, NEFL, CTSH, FLNB, PDLIM5, BCAP31, RHOG, VAMP8, FABP4, ABI2, FKBP1B, ALOX5AP, ATP6V1B2, IRF8, AMOTL2 |  |
| CC | GO:0042470 | melanosome | 0.025518 | PRDX1, ATP6V1B2, TPP1, CAPG |  |
| CC | GO:0010008 | endosome membrane | 0.028955 | HLA-DPB1, CD14, HLA-DOA, HLA-DQB2, HLA-DPA1 |  |
| CC | GO:0016021 | integral component of membrane | 0.029696 | COX4I1, SLC40A1, SLC1A3, CXCR4, SLC2A5, HLA-DMA, HLA-DMB, SYNGR2, SV2B, ADAMTS1, CDH1, FCGRT, PODXL, C3AR1, FLNB, HLA-DOA, HLA-DPA1, CD53, COX8A, CCR1, BCAP31, CD74, FCER1G, PAQR3, NPY1R, UNC5C, TGFBR3, VAMP8, BTC, MS4A6A, TYROBP, SLC7A7, MMP13, RNF149, ALOX5AP, ATP6V1B2, HLA-DPB1, SLC25A5, FCGR2B, PAM, ITM2A, HLA-DQB2, SLC25A6 |  |
| CC | GO:0031012 | extracellular matrix | 0.038397 | MMP13, PRDX1, LTBP2, FLNB, SLC25A5, SLC25A6 |  |
| CC | GO:0005938 | cell cortex | 0.042027 | CLIC5, MARCKS, FLNB, CTGF |  |
| CC | GO:0005576 | extracellular region | 0.042765 | PCOLCE2, COL22A1, PLEK, F13A1, IFI30, MMP9, CTGF, TGFBR3, BTC, MMP13, C7, CDH1, CTSL, SPP1, CCL2, CD14, HBEGF |  |
| CC | GO:0033180 | proton-transporting V-type ATPase, V1 domain | 0.048971 | ATP6V1A, ATP6V1B2 |  |
| MF | GO:0032395 | MHC class II receptor activity | 2.29E-06 | HLA-DMA, HLA-DPB1, HLA-DOA, HLA-DQB2, HLA-DPA1 |  |
| MF | GO:0023026 | MHC class II protein complex binding | 1.46E-04 | CD74, HLA-DMA, HLA-DMB, HLA-DOA |  |
| MF | GO:0005518 | collagen binding | 6.50E-04 | MMP13, PCOLCE2, CTSL, MMP9, DDR2 |  |
| MF | GO:0008201 | heparin binding | 6.59E-04 | TGFBR3, PCOLCE2, ADAMTS1, CCL2, LTBP2, CTGF, HBEGF |  |
| MF | GO:0019864 | IgG binding | 0.002267 | FCER1G, FCGRT, FCGR2B |  |
| MF | GO:0005515 | protein binding | 0.004176 | CLIC5, DOCK4, DOCK9, COX4I1, PLEK, FHL1, SLC40A1, SNX10, IFI30, CTGF, FRAT1, CDH1, CTSL, AP1S2, COTL1, NEFL, CTSH, HLA-DOA, CTSC, SOX5, HTATIP2, CD53, COX8A, PDGFRA, FCER1G, RHOG, NPY1R, MMP9, TGFBR3, BTC, VAMP8, TYROBP, ATP6V1B2, IRF8, NCOA7, SLC25A5, SMPDL3A, SLC25A6, DDR2, GRN, CXCR4, CAPG, LTBP2, NPL, FYB, SOCS2, ABLIM1, SYNGR2, SV2B, CALD1, PODXL, PRDX1, SPP1, FLNB, CD14, CCL18, PDLIM5, MPP1, BCAP31, CCR1, TCF7L2, CD74, LAPTM5, KLF4, MAFB, ABI2, FKBP1B, ALOX5AP, TPP1, AMOTL2, FCGR2B, PAM, ITM2A |  |
| MF | GO:0046934 | phosphatidylinositol-4,5-bisphosphate 3-kinase activity | 0.007898 | BTC, PDGFRA, PIK3R5, HBEGF |  |
| MF | GO:0003779 | actin binding | 0.010143 | ABLIM1, CALD1, COTL1, CXCR4, CAPG, FLNB, PDLIM5 |  |
| MF | GO:0098641 | cadherin binding involved in cell-cell adhesion | 0.012304 | CALD1, DOCK9, CDH1, PRDX1, CAPG, FLNB, PDLIM5 |  |
| MF | GO:0042605 | peptide antigen binding | 0.014489 | FCGRT, HLA-DPB1, HLA-DPA1 |  |
| MF | GO:0015207 | adenine transmembrane transporter activity | 0.019598 | SLC25A5, SLC25A6 |  |
| MF | GO:0008234 | cysteine-type peptidase activity | 0.025825 | CTSL, CTSH, CTSC |  |
| MF | GO:0004252 | serine-type endopeptidase activity | 0.026614 | MMP13, CTSL, CTSH, TPP1, MMP9, CTSC |  |
| MF | GO:0005080 | protein kinase C binding | 0.036757 | MARCKS, PLEK, PDLIM5 |  |
| MF | GO:0015171 | amino acid transmembrane transporter activity | 0.038229 | SLC7A7, SLC1A3, SLC7A2 |  |
| MF | GO:0015174 | basic amino acid transmembrane transporter activity | 0.038815 | SLC7A7, SLC7A2 |  |
| MF | GO:0005088 | Ras guanyl-nucleotide exchange factor activity | 0.040209 | BTC, PDGFRA, NEFL, HBEGF |  |
| MF | GO:0004175 | endopeptidase activity | 0.049137 | CTSH, TPP1, MMP9 |  |

Abbreviation: GO, gene ontology. DEGs, differentially expressed genes. PVNS, pigmented villous nodular synovitis. BP, biological process. CC, cellular component. MF, molecular function.

Table S2: KEGG pathways analysis of DEGs associated with PVNS

| **Pathway** | **ID** | **P value** | **Genes** |
| --- | --- | --- | --- |
| Rheumatoid arthritis | hsa05323 | 2.57E-06 | ATP6V1A, HLA-DMA, HLA-DMB, CTSL, HLA-DPB1, ATP6V1B2, CCL2, HLA-DOA, HLA-DPA1 |
| Antigen processing and presentation | hsa04612 | 1.04E-05 | CD74, HLA-DMA, HLA-DMB, CTSL, HLA-DPB1, IFI30, HLA-DOA, HLA-DPA1 |
| Asthma | hsa05310 | 1.10E-05 | HLA-DMA, HLA-DMB, FCER1G, HLA-DPB1, HLA-DOA, HLA-DPA1 |
| Staphylococcus aureus infection | hsa05150 | 1.57E-05 | HLA-DMA, HLA-DMB, HLA-DPB1, C3AR1, FCGR2B, HLA-DOA, HLA-DPA1 |
| Phagosome | hsa04145 | 1.83E-05 | ATP6V1A, HLA-DMA, HLA-DMB, CTSL, HLA-DPB1, ATP6V1B2, CD14, FCGR2B, HLA-DOA, HLA-DPA1 |
| Intestinal immune network for IgA production | hsa04672 | 1.04E-04 | HLA-DMA, HLA-DMB, HLA-DPB1, CXCR4, HLA-DOA, HLA-DPA1 |
| Graft-versus-host disease | hsa05332 | 3.22E-04 | HLA-DMA, HLA-DMB, HLA-DPB1, HLA-DOA, HLA-DPA1 |
| Tuberculosis | hsa05152 | 3.97E-04 | CD74, HLA-DMA, HLA-DMB, FCER1G, HLA-DPB1, CD14, FCGR2B, HLA-DOA, HLA-DPA1 |
| Allograft rejection | hsa05330 | 5.04E-04 | HLA-DMA, HLA-DMB, HLA-DPB1, HLA-DOA, HLA-DPA1 |
| Type I diabetes mellitus | hsa04940 | 8.22E-04 | HLA-DMA, HLA-DMB, HLA-DPB1, HLA-DOA, HLA-DPA1 |
| Autoimmune thyroid disease | hsa05320 | 0.001843 | HLA-DMA, HLA-DMB, HLA-DPB1, HLA-DOA, HLA-DPA1 |
| Viral myocarditis | hsa05416 | 0.002588 | HLA-DMA, HLA-DMB, HLA-DPB1, HLA-DOA, HLA-DPA1 |
| Inflammatory bowel disease (IBD) | hsa05321 | 0.003947 | HLA-DMA, HLA-DMB, HLA-DPB1, HLA-DOA, HLA-DPA1 |
| HTLV-I infection | hsa05166 | 0.004027 | PDGFRA, HLA-DMA, HLA-DMB, HLA-DPB1, SLC25A5, HLA-DOA, SLC25A6, HLA-DPA1, PIK3R5 |
| Leishmaniasis | hsa05140 | 0.005723 | HLA-DMA, HLA-DMB, HLA-DPB1, HLA-DOA, HLA-DPA1 |
| Lysosome | hsa04142 | 0.007546 | CTSL, AP1S2, LAPTM5, CTSH, TPP1, CTSC |
| Influenza A | hsa05164 | 0.008264 | HLA-DMA, HLA-DMB, HLA-DPB1, CCL2, HLA-DOA, HLA-DPA1, PIK3R5 |
| Herpes simplex infection | hsa05168 | 0.01046 | CD74, HLA-DMA, HLA-DMB, HLA-DPB1, CCL2, HLA-DOA, HLA-DPA1 |
| Systemic lupus erythematosus | hsa05322 | 0.011454 | HLA-DMA, HLA-DMB, C7, HLA-DPB1, HLA-DOA, HLA-DPA1 |
| Cell adhesion molecules (CAMs) | hsa04514 | 0.014446 | HLA-DMA, HLA-DMB, CDH1, HLA-DPB1, HLA-DOA, HLA-DPA1 |
| Toxoplasmosis | hsa05145 | 0.025325 | HLA-DMA, HLA-DMB, HLA-DPB1, HLA-DOA, HLA-DPA1 |
| Axon guidance | hsa04360 | 0.039912 | ABLIM1, SEMA3C, SEMA3B, CXCR4, UNC5C |

Abbreviation: KEGG, Kyoto Encyclopedia of Genes and Genomes. DEGs, differentially expressed genes. PVNS, pigmented villous nodular synovitis.
